# Imaging features of the postoperative spine: a guide to basic understanding of spine surgical procedures

**DOI:** 10.1186/s13244-023-01447-0

**Published:** 2023-06-06

**Authors:** Marília Maria Vasconcelos Girão, Lucas Kenzo Miyahara, Viviane Sayuri Yamachira Dwan, Eduardo Baptista, Atul Kumar Taneja, Alberto Gotfryd, Adham do Amaral e Castro

**Affiliations:** 1 Hospital Israelita Albert Einstein, São Paulo, Brazil; 2 Federal University of São Paulo, Rua Napoleão de Barros, n° 800, São Paulo, 04024-002 Brazil; 3 Department of Radiology, UT Southwestern Medical Center, Dallas, TX USA

**Keywords:** Spine, Postoperative complications, Multimodal imaging, Orthopedic hardware

## Abstract

**Abstract:**

Spinal surgical procedures are becoming more common over the years, and imaging studies can be requested in the postoperative setting, such as a baseline study when implants are used, or when there is a new postoperative issue reported by the patient or even as routine surveillance. Therefore, it helps the surgeon in the appropriate management of cases. In this context, there is increasing importance of the radiologist in the adequate interpretation of postoperative images, as well as in the choice of the most appropriate modality for each case, especially among radiographs, computed tomography, magnetic resonance imaging and nuclear medicine. It is essential to be familiar with the main types of surgical techniques and imaging characteristics of each one, including the type and correct positioning of hardware involved, to differentiate normal and abnormal postoperative appearances. The purpose of this pictorial essay is to illustrate and discuss the more frequently used spine surgical interventions and their imaging characteristics, with an emphasis on classical decompression and fusion/stabilization procedures.

**Graphical Abstract:**

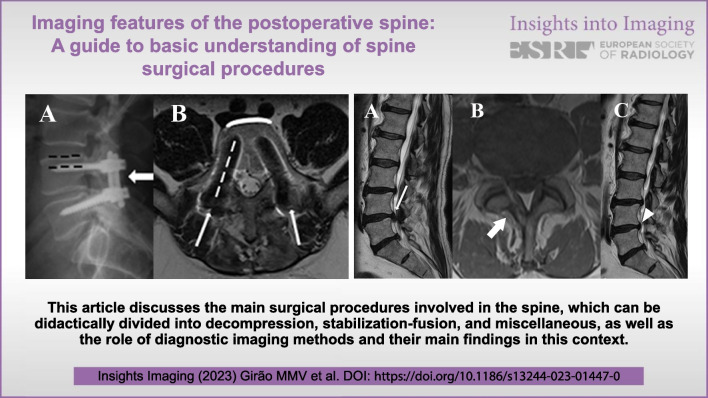

**Key points:**

- Plain radiographs remain the main modality for baseline, dynamic evaluation, and follow-ups.
- CT is the method of choice for assessing bone fusion, hardware integrity and loosening.
- MRI should be used to evaluate bone marrow and soft tissue complications.
- Radiologists should be familiar with most performed spinal procedures in order to differentiate normal and abnormal.

**Critical relevance statement:**

This article discusses the main surgical procedures involved in the spine, which can be didactically divided into decompression, stabilization-fusion, and miscellaneous, as well as the role of diagnostic imaging methods and their main findings in this context.

## Background

Spinal surgeries are among the most frequently performed orthopedic-neurosurgical procedures [1] and part of the routine of the general radiologist, and especially within musculoskeletal and neuroradiology specialties.

The postoperative evaluation of spine is sometimes complex and time-consuming, with imaging being essential for the management of such patients [2]. It is useful for the radiologist to be aware of surgical indications, types of procedures performed, time elapsed since surgery and current symptoms [3]. In this manner, it will become easier to interpret and differentiate normal from abnormal postoperative findings, as well as to prepare a clear and useful report to the surgeon.

This pictorial essay provides an overview of basic spine surgical interventions, as well as the hardware involved and the adequate imaging modality to assess each procedure.

## Imaging modalities

### Plain radiographs (X-ray)

**Fig. 1:**
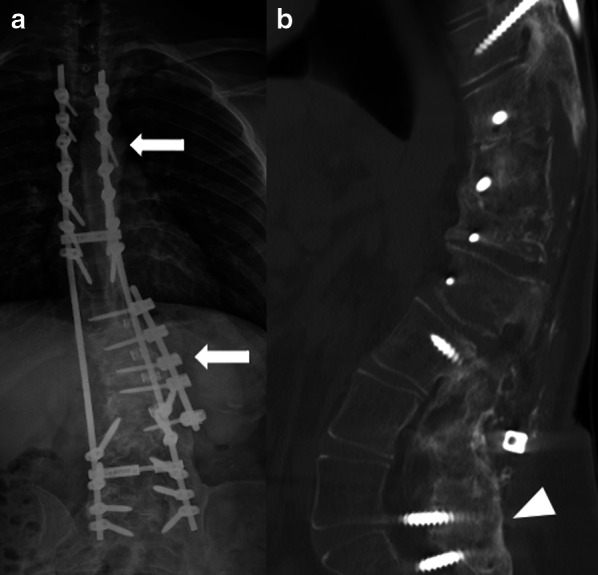
Stabilization construct for deformity correction (scoliosis). Anteroposterior (**a**) radiograph and sagittal CT reformat in bone algorithm (**b**) demonstrate a fusion/stabilization procedure using bilateral rods with transpedicular and lateral body screws (arrows) extending through the thoracolumbar spine (from T4-T5 to L5-S1). There is no sign of loosening (such as lucency around the screws) or fracture of components. Note the fusion (arthrodesis) of the posterior elements of the lumbar spine (arrowhead), indicating a successful outcome

Plain radiographs are the most frequent modality used for postoperative spine evaluation due to its easy access and cost-effectiveness [3], usually including anteroposterior (AP), lateral and flexion–extension motion views. It is important to perform an immediate postoperative baseline radiograph to serve as a comparison parameter for future studies [4]. Radiographs allow an overview of the relationship between surgical instruments and bone structures (Fig. 1A), evaluating complications related to the implants, fusion status, and alignment. It can also assess instability by dynamic acquisition [1].

Panoramic radiographs play a major role in the preoperative evaluation for the detection of scoliosis, malalignment, vertebral counting, and assessment of transitional vertebrae to ensure that the surgery occurs at the correct level, serving also as a reference for intraoperative fluoroscopy, especially to check spine levels and confirm appropriate position and location of implants [1, 2, 4].

Reasons to perform postoperative imaging include reassuring the patient about adequate healing and surgery success, identifying asymptomatic hardware migration or failure, and documenting clinical status in the medical record [5].

### Computed tomography (CT)

CT is the most accurate modality for assessing mineralized bone structures, including evaluation of spine fusion progression (Fig. 1B), which is expected to occur within 9 months after surgery [4], and it is better assessed by coronal and sagittal CT reformats. CT is also helpful to evaluate hardware-related complications, such as malposition, failure, or loosening. Beam hardening artifacts may obscure the abnormalities, although artifact reduction software [1, 4, 6, 7] can aid to reduce this issue. CT myelography can be used as an alternative to evaluate the exiting nerve roots and spinal canal in patients with restriction to magnetic resonance imaging (MRI) [8, 9].

### MRI

MRI is the most reliable choice to assess postoperative complications due to its excellent soft-tissue resolution [4]. It allows the evaluation of the vertebral canal, nerve roots, bone marrow, and paravertebral soft tissues.

**Fig. 2:**
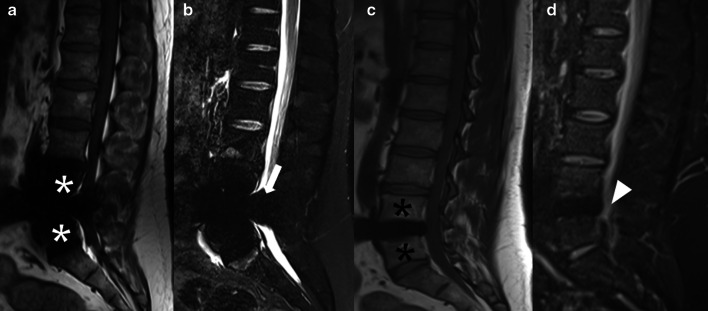
Technique for metal artifacts reduction after a disc replacement procedure at the L4-5 level. Sagittal T1-weighted (**a**) and STIR (**b**) MR images show susceptibility artifacts due to the metallic disc prosthesis, limiting the evaluation of the vertebral bodies (white asterisks) and spinal canal (arrow) from L3-4 to L5-S1 levels at these levels. Sagittal T1-weighted (**c**) and STIR (**d**) MAVRIC sequences to reduce metal artifacts improve the evaluation of the L3-4 and L5-S1 levels (black asterisks), and the spinal canal (arrowhead) and posterior elements

Although there are no major concerns regarding spinal fusion-hardware for the safety of the patient undergoing an MR procedure, they can still significantly impair the evaluation of MRI images due to magnetic susceptibility artifacts [10]. Some alternatives to reduce these artifacts include increasing the bandwidth, higher matrix size and/or the turbo factor, lower magnetic field, acquisition in fast spin echo and short tau inversion recovery (STIR), and techniques for fat suppression [3]. New techniques are currently available to reduce artifacts [11] (Fig. 2) and significantly improve image quality [12], including multi-acquisition variable resonance image combination selective (MAVRIC-SL, GE HealthCare^®^), WARP (Siemens Healthineers^®^), and slice encoding metal artifact correction (SEMAC, Siemens Healthineers^®^) [13].

### Nuclear medicine

Nuclear medicine studies are also part of the diagnostic workup of the postoperative spine. These examinations are typically used in certain situations, such as in patients in whom MRI is contraindicated or if MRI and CT are equivocal. The most important studies in this setting are gallium scan, indium 111-tagged white blood cell (WBC) scan, single-photon emission computerized tomography/computed tomography (SPECT/CT), and positron emission tomography (PET) scan [14–16].

Ga-67 scintigraphy combined with SPECT/CT can be used to evaluate suspected spinal infection in patients who have undergone recent spine interventions. Although Ga-67 is less sensitive (73%), it is more specific (61%) than skeletal scintigraphy. Moreover, a dual Ga-67 and Tc-99m-MDP study can increase the overall specificity of the examination to 81% with a sensitivity of 73% [14].

SPECT/CT can provide information on metabolic activity with anatomical localization and may identify postoperative complications like pseudarthrosis, radiculopathy, adjacent segment degeneration, hardware failure, and radiographically occult fractures [5]. Overall, SPECT/CT has a high sensitivity and can assess osteoblast activity; however, it lacks specificity and is unable to assess disc herniation, root compression, or stenosis. SPECT/CT can be used as part of the diagnostic workup of suspected early posterior operative complications or recurrent pain posterior to surgery [15].

**Fig. 3:**
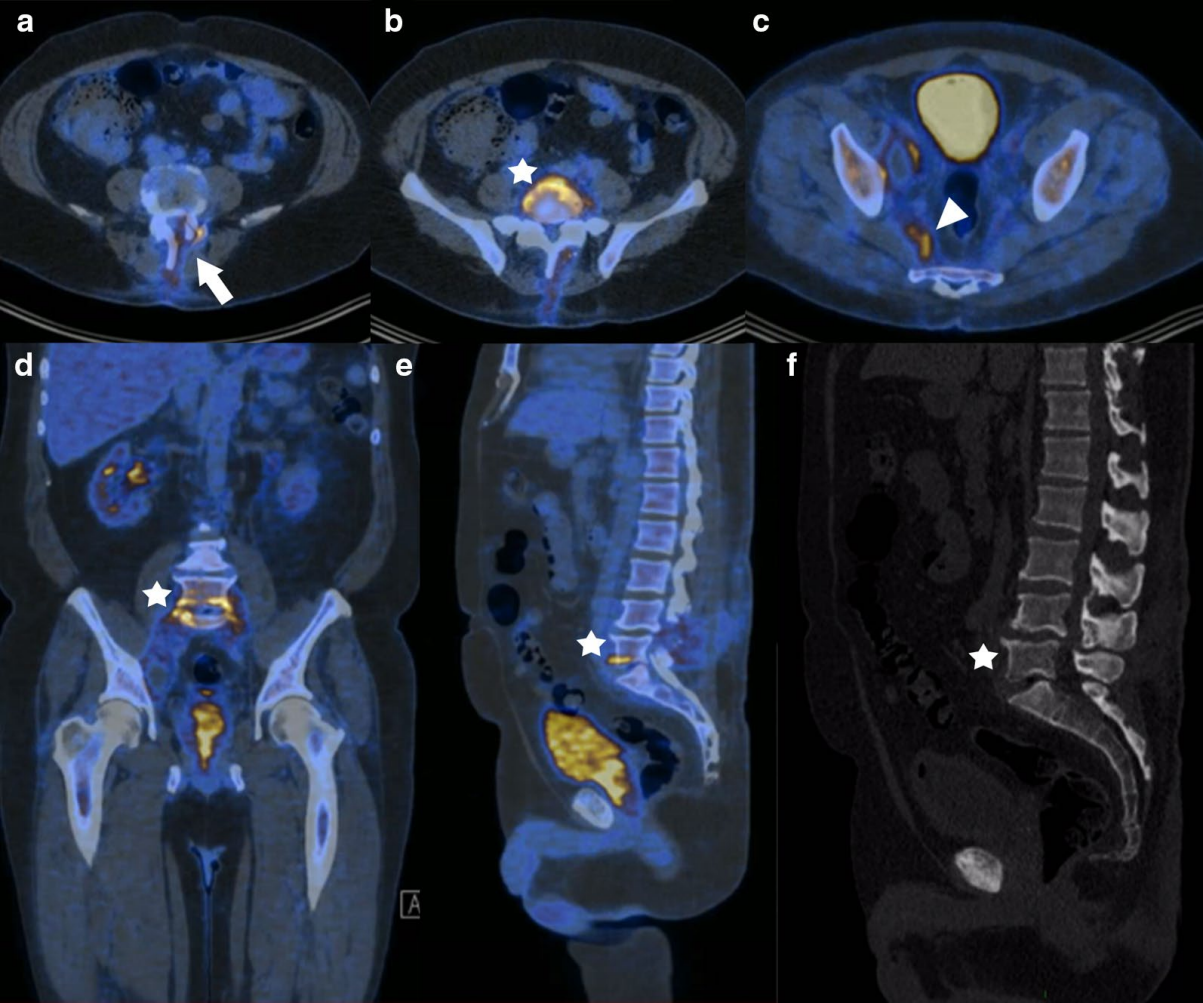
PET-CT in the setting of infection. PET-CT shows expected metabolic activity at the site of recent surgical intervention (laminectomy) (arrow) of the lumbar spine (**a**). Increased metabolism (**b**, **d** and **e**) associated with cortical irregularities and foci of osteolysis (**f**) are observed in the anterior aspect of L5 and S1 endplates (stars), consistent with ongoing inflammatory/infectious process. It is also observed a small fluid collection with increased metabolism in the right aspect of the pelvic cavity (**c**) (arrowhead)

PET using the fluorine-18-2-fluoro-2-deoxy-D-glucose (FDG) tracer with CT has gained a lot of role in post-operative assessment, especially in cases where infection is suspected [14]. Studies have shown the utility of this modality in the initial evaluation of potentially infected spinal implants (Fig. 3) [16]. However, it remains indicated for cases in which the MRI is inconclusive [14].

## Surgical procedures

The most common indications for spinal surgery are refractory back or neck pain and neurologic deficit related to nerve root or spinal cord compression, especially related to degenerative disc disease. Surgical technique, incision approach and hardware chosen will vary depending on the anatomic features of each segment, surgery indication and surgeon's experience [1].

Spinal surgeries can be didactically classified into three main categories: (1) decompressive; (2) stabilization-fusion (including those for deformity correction); and (3) miscellaneous procedures [2, 4, 17]. In many cases, these procedures are used concurrently.

### Decompressive procedures

These techniques are performed to remove compressing factors to the nerve roots and/or the spinal cord. Such compression may be caused by a herniated disc, hypertrophic facets (bone spurs), synovial cysts, thickened *ligamentum flavum*, tumors, abscesses, or hematomas [1, 18].

The most used lumbar decompression techniques, along with discectomy, are laminotomy and laminectomy (with or without medial facetectomy) [2]. The choice of the technique depends on the type, location, and size of the compressive element [19].

Minimally invasive surgeries, including endoscopic and microscopic tubular surgeries, are becoming more frequent, with microdiscectomies being performed for small disc herniations, occasionally with minimal resection of posterior elements. These characteristics lead to a challenge in recognition of these surgeries through images, especially after a long postoperative time [9].

**Fig. 4:**
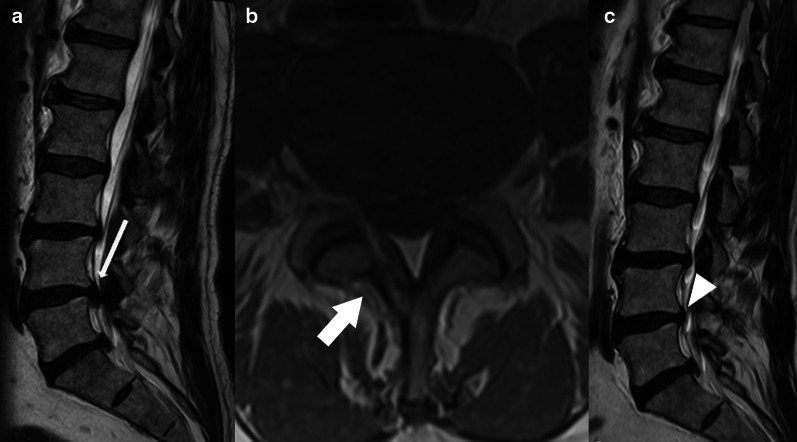
Discectomy and laminotomy. Preoperative T2W sagittal MR image (**a**) shows the herniated and inferiorly migrated disc at the L4-5 level, abutting the anterior thecal sac (thin arrow) and right L5 nerve root (not shown). There is also a thickened ligamentum flavum. Postsurgical T1W axial (**b**) and T2W sagittal MR images (**c**) at the same level as “(**a**)” demonstrates right laminotomy (arrow) with removal of herniated disc material—discectomy (arrowhead)

**Fig. 5:**
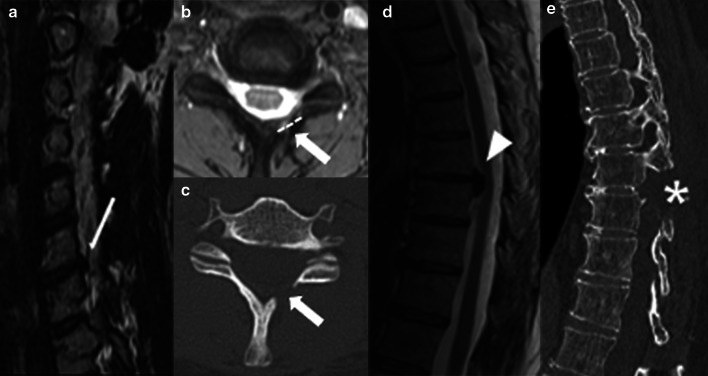
Discectomy and laminectomy. A disc herniation (thin arrow) at the C6-7 level is demonstrated on sagittal T2W preoperative MR image (**a**). Discectomy with laminectomy and flavectomy (arrows) were performed, as shown on axial GRE MRI (**b**) and CT image in bone window (**c**). Note that there is a larger resection area (dashed line) compared to laminotomy (Fig. 3B). A larger disc herniation (arrowhead) in another patient at the T8-9 level is shown on a sagittal T2W preoperative MR image (**d**). To gain a greater exposure to the vertebral canal, a discectomy using the laminectomy with medial facetectomy technique (asterisk) was performed, as demonstrated on the sagittal CT reformat (**e**). This procedure comprises in removal of the lamina and a partial resection of the facet joint

- *Laminotomy or “laminae fenestration”* (Fig. 4). Resection of a small inferior segment of the upper level lamina and sometimes the superior segment of the lower level lamina [3, 4]. The lamina fenestration can be performed bilaterally by a unilateral approach through resection of the base of the spinous process, a technique known as “over the top” decompression.
- *Laminectomy/Laminectomy with medial facetectomy* (Fig. 5). Complete resection of the lamina and *ligamentum flavum* (flavectomy), which can be unilateral or bilateral. When bilateral (or total), it also involves the removal of the spinous process. In order to gain maximum exposure to the spinal canal and foramina, medial facetectomy can be associated, but it is important to preserve the facet joint as much as possible to prevent instability [3, 4]. The resection of more than 50% of a facet joint may lead to mechanical instability. It may be challenging to identify laminectomy at MRI and a hint that can be used is searching for the flavectomy site.

### Stabilization-fusion procedures

The goal of these techniques is to maintain correct alignment and prevent abnormal motion of spine. They are also used to replace excised components of the spine. Stabilization is achieved by fusion of vertebral bodies and/or posterior elements through instrumentation and bone graft, which provide support until bone fusion (arthrodesis) occurs [1, 20].

Indications include degenerative disc disease, deformity correction, spondylolisthesis, tumors, trauma, and infection [2].

Although it might be challenging for the radiologist to remember the name of all devices used in spine surgery, it is possible to learn the treatment principles and basic categories of the hardware based upon their objectives [1].

The cervical spine is approached anteriorly and posteriorly, while the thoracic spine is more often approached posteriorly.

The lumbar spine may be manipulated anteriorly (also named anterior lumbar interbody fusion—ALIF), laterally (extreme lateral interbody fusion—LLIF) and posteriorly (posterior lumbar interbody fusion—PLIF). Alternative approaches are posterior transforaminal (transforaminal lumbar interbody fusion—TLIF) and a posterolateral intertransverse (intertransverse lumbar interbody fusion—ILIF).

**Fig. 6:**
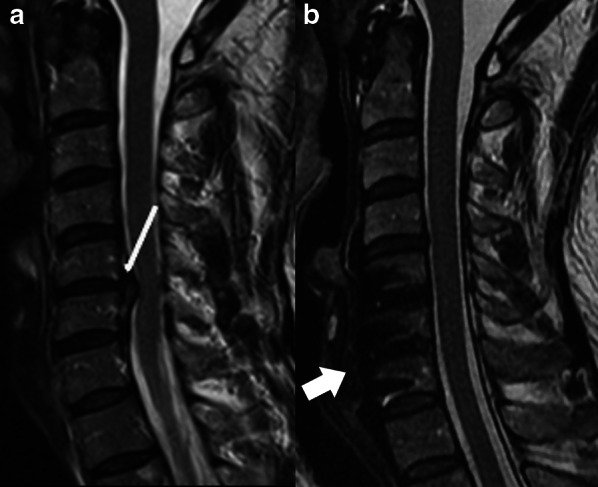
Plate with screws—anterior fusion procedure for degenerative disc disease. A disc herniation at the C5-6 level (thin arrow) compressing the spinal cord is demonstrated on a preoperative T2W sagittal MR image (**a**). An anterior cervical fusion procedure was performed from C5 to C7 using plate with screws attached to the vertebral bodies (arrow), as shown on a postsurgical T2W sagittal MR image (**b**). Resection of the herniated disc material was performed

**Fig. 7:**
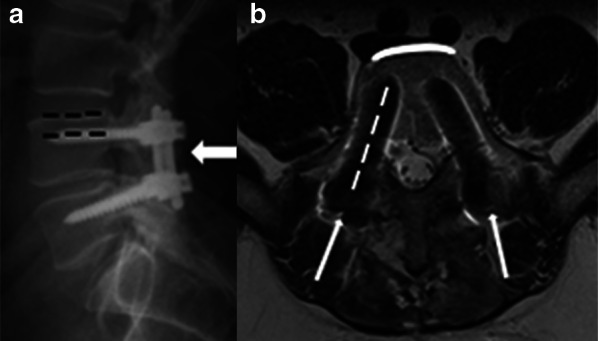
A typical arthrodesis with rod and transpedicular screws—posterior approach for degenerative disc disease. Lateral radiography (**a**) and T2W axial MR image (**b**) after a fusion procedure using stabilization hardware consisting of bilateral rods and transpedicular screws at the L4-5 intervertebral level (arrow). There is optimal pedicle screw positioning. The screws traverse the pedicle without lateral or medial angulation (thin arrow and white dashed line) and the anterior vertebral body cortex is intact (curved line). The screws are also parallel to the vertebral body endplate in the lateral/sagittal plane (black dashed line)

**Fig. 8:**
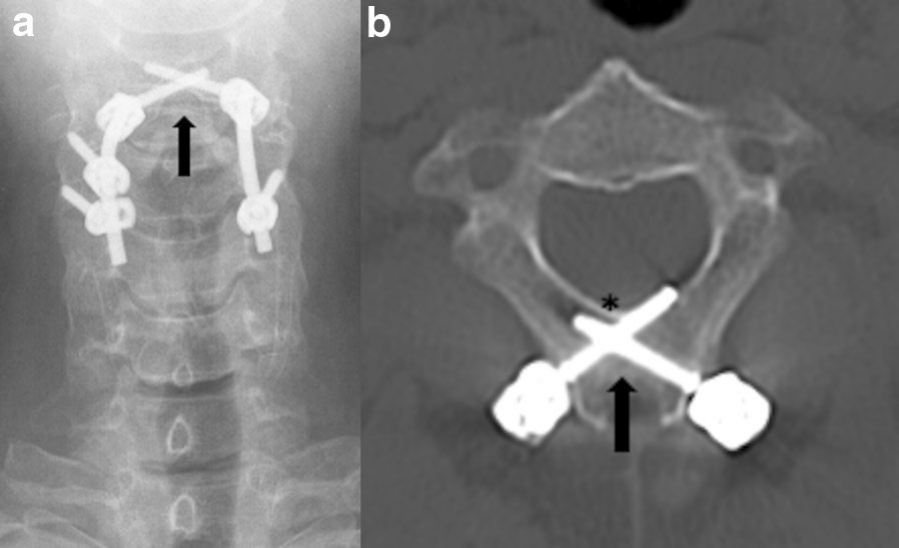
Rods with translaminar screws—posterior stabilization procedure due to trauma (fracture and a mild traumatic anterolisthesis). Anteroposterior radiograph (**a**) and axial CT image (**b**) demonstrate bilateral crossing C2 laminar screws (black arrows). Although the right screw is tangent to the inner cortex (asterisk), this positioning is acceptable

**Fig. 9:**
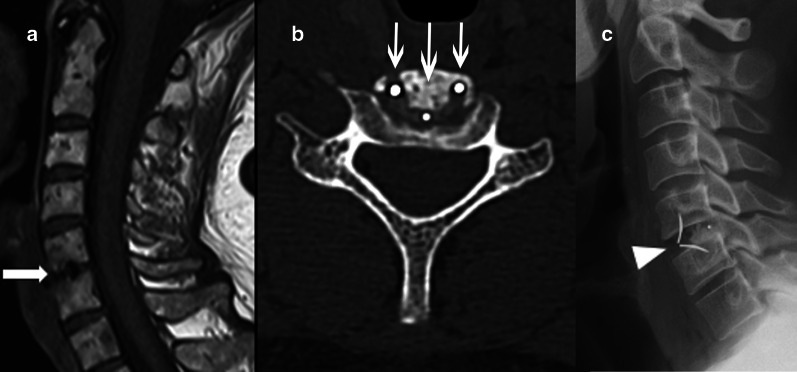
Anterior fusion procedure with interbody cage spacer due to trauma (**a** and **b**) and degenerative disc disease (**c**). Sagittal T1W MR image (**a**) demonstrates mild magnetic susceptibility artifact produced by the metallic cage (arrow) at the C5-6 level, making it difficult to evaluate. Axial CT image (**b**) demonstrates optimal placement of the interbody spacer in the center of the vertebral body, as delineated by radiopaque markers (thin arrows). A posterior marker located at least 2 mm anterior to the posterior edge of the adjacent vertebral body indicates the spacer is in a good position. Lateral radiograph (**c**) from another patient demonstrates a stand-alone cage with self-locking plates (arrowhead) attached to the vertebral bodies to provide stability. The spacers’ purpose is to promote fusion while maintaining spine alignment and support

The most used hardware for these techniques are:

- *Plate with screws attached to the vertebral body* (Fig. 6). The anterior approach for degenerative disc disease is frequently used in the cervical spine. Lateral elements screw fixation is used by a posterior approach.
- *Rods with transpedicular screws* (Fig. 7). Posterior approach for degenerative disc disease is frequently used anywhere from thoracic spine to the sacrum. The screws must reach the vertebral bodies through the pedicles, where they must be centralized. Also, screw ends should not extend beyond the anterior vertebral body cortex or trespassing the endplates [2, 21]. Ideally, they should be placed parallel to the vertebral body endplate in the sagittal plane. In case when deformity correction (scoliosis) is needed, the surgery often requires using large constructions and multiple approaches (Fig. 1).
- *Translaminar screws* (Fig. 8). An alternative and less invasive posterior fusion technique compared to the transpedicular screws used in the cervical spine [22, 23]. A C2 translaminar screw fixation is called Wright’s/modified Wright’s technique, and it is relevant in cases of atlantoaxial instability [24].
- *Interbody cage spacers* (Fig. 9). Device placed in the intervertebral space to restore disc height and facilitate bone growth to promote intervertebral fusion [2, 7]. Bone graft is often used to fill the cage spacer and around it to stimulate ossification. The position of the cages can be assessed using radiopaque markers, and the posterior marker should be no less than 2 mm from the posterior edge of the adjacent vertebral body [20]. It is called stand-alone interbody fusion when the cage is affixed directly to the vertebral bodies without additional surgical instrumentation [2].

### Miscellaneous procedures

In this category, we will include non-classical procedures used for varied purposes, including surgery with dynamic non-fusion systems (disc arthroplasty and techniques with posterior motion preservation devices, for example), lesion excision procedures and vertebral body replacement.

**Fig. 10:**
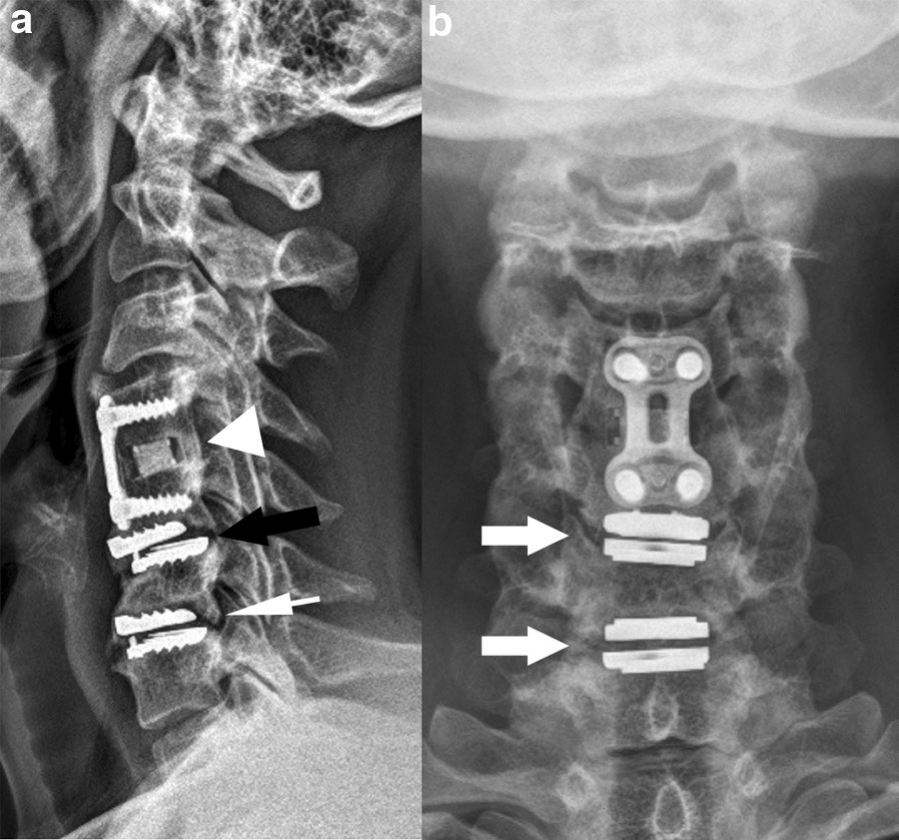
Total disc replacement (or disc arthroplasty). Lateral (**a**) and anteroposterior (**b**) radiographs demonstrate disc arthroplasty at C5-6 and C6-7 levels. There is also an anterior fusion/stabilization hardware with plates, screws, and an interbody spacer (arrowhead) at the C4-5 level. Disc prostheses (arrows) are composed of a core sandwiched between two metal plates that attach to the vertebral bodies. Ideally, the plates must be at the center of the vertebral body and parallel to each other. In this case, there is a slight anterior dislocation of the superior plate at C5-6 level (black arrow) and optimal positioning at the C6-7 level (thin arrow)

**Fig. 11:**
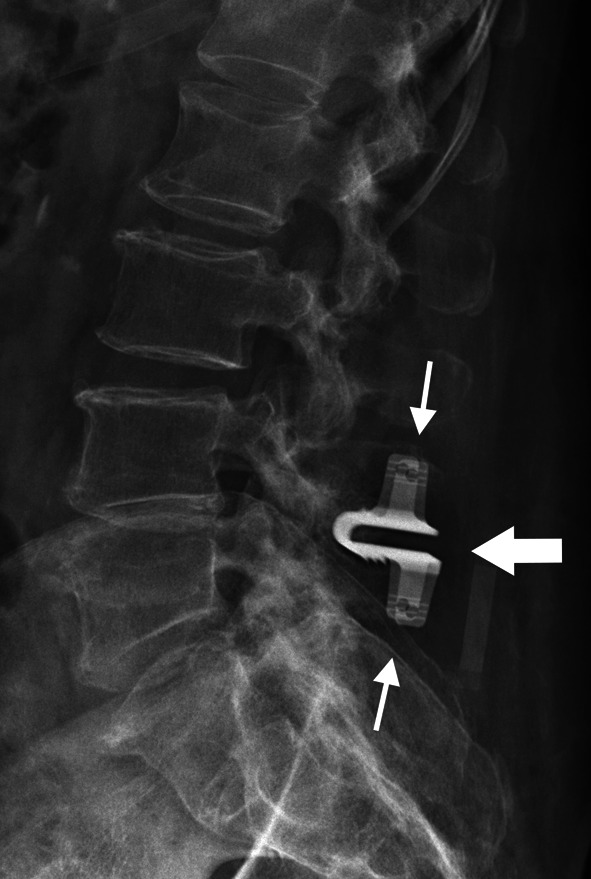
Dynamic stabilization system with interspinous spacer for intermittent neurogenic claudication. Lateral radiograph demonstrates the U-shaped device (arrow) placed into the interspinous space at the symptomatic level (L4-5). To maintain stability, the vertical keels are crimped onto the spinous processes (thin arrow). This procedure requires resection of both interspinous and supraspinous ligaments. A common complication in such procedures is spinous process resorption along the implant close to the fixation site. There are other devices available for the same proposal with less invasive approaches

**Fig. 12:**
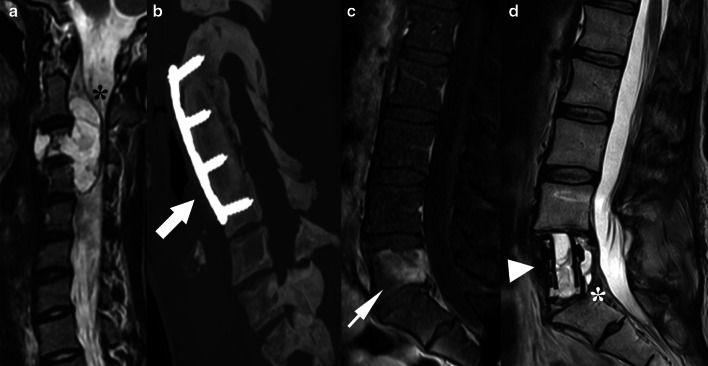
Vertebral body replacement (corpectomy). Sagittal T2W preoperative MR image (**a**) shows an expansile intracanal chordoma (black asterisk) centered in C3 vertebral body and extending from C2 to C4 levels. The patient underwent vertebral bodies replacement (corpectomy) and anterior fusion procedure using plate and screws (arrow), with interposition of bone graft, as shown on a postoperative sagittal using maximum intensity projection CT reconstruction in bone window (**b**). In another patient, sagittal T1W fat-suppressed post-contrast MR image (**c**) presents a spinal metastasis from breast cancer involving the L5 vertebral body (thin arrow). Postoperative sagittal T2W MR image (**d**) demonstrates extensive resection of L5 vertebral body (corpectomy) with placement of a spacer (arrowhead). There is a small amount of fluid around the spacer (white asterisk), in this case related to postoperative seroma that was later absorbed

- *Disc arthroplasty or total disc replacement* (Fig. 10). An alternative procedure to the anterior fusion which attempts to preserve the normal motion and biomechanics of the spine, reducing the incidence of adjacent-level degeneration [8, 20, 23]. There must be more than 4 mm of residual disc height and no significant degeneration of the end plate and neither the facet joint [8, 25]. The procedure involves discectomy, excision of anterior and posterior longitudinal ligaments, and placement of discal prosthesis composed of a core sandwiched between two plates attached to the vertebral bodies [2, 26].
- *Dynamic stabilization with interspinous spacer* (Fig. 11). Indicated for the treatment of a position-dependent intermittent neurogenic claudication related to spinal stenosis, as this distraction device mimics a flexed position and decreases the facet load [6, 20, 26]. The procedure is less invasive when compared to conventional fusion and preserves the longitudinal ligaments intact [26]. However, the long-term effectiveness of these devices has yet to be established.
- *Lesion excision procedures*. Include many different procedures, such as infection debridement, abscess drainage and tumor resection (Fig. 12), each one with its own features.
- *Vertebral body replacement/corpectomy* (Fig. 12). Performed in cases where there is an extensive removal of the vertebral body, secondary to tumor involvement or trauma, requiring a replacement procedure that can use different devices to maintain spine stability [2].

## Conclusion and take-home messages

Since the evaluation of the postoperative spine is challenging, it is of utmost importance to have a basic understanding of indications and objectives of the main surgical procedures, as well as the hardware instrumentation materials and their normal appearance through imaging. One should be familiar with the most appropriate imaging modality for each setting, including radiographs, CT, MRI, or nuclear medicine studies. Comparison with previous exams and access to surgical descriptions are always helpful and support imaging evaluation. The ultimate goal is to create efficient reports to assist the surgeon in continuous patient care.

## Data Availability

Data sharing is not applicable to this article as no datasets were generated or analyzed during the current study.

## Abbreviations

ALIF: Anterior lumbar interbody fusion
AP: Anteroposterior
CT: Computed tomography
FDG: Fluorine-18-2-fluoro-2-deoxy-D-glucose
ILIF: Intertransverse lumbar interbody fusion
LLIF: Extreme lateral interbody fusion
MAVRIC-SL: Multi-acquisition variable resonance image combination selective
MRI: Magnetic resonance imaging
PET: Positron emission tomography
PLIF: Posterior lumbar interbody fusion
SEMAC: Slice encoding metal artifact correction
SPECT/CT: Single-photon emission computerized tomography/computed tomography
STIR: Short tau inversion recovery
TLIF: Transforaminal lumbar interbody fusion
WBC: White blood cell
